# Prediction of Retention Time and Collision Cross Section (CCS_H+_, CCS_H–_, and CCS_Na+_) of Emerging Contaminants Using Multiple Adaptive Regression Splines

**DOI:** 10.1021/acs.jcim.2c00847

**Published:** 2022-10-24

**Authors:** Alberto Celma, Richard Bade, Juan Vicente Sancho, Félix Hernandez, Melissa Humphries, Lubertus Bijlsma

**Affiliations:** † Environmental and Public Health Analytical Chemistry, Research Institute for Pesticides and Water, University Jaume I, E-12071Castelló, Spain; ‡ Department of Aquatic Sciences and Assessment, Swedish University of Agricultural Sciences (SLU), SE-750 07Uppsala, Sweden; § University of South Australia, Adelaide, UniSA: Clinical and Health Sciences, Health and Biomedical Innovation, AdelaideSA-5000, South Australia, Australia; ∥ Queensland Alliance for Environmental Health Sciences (QAEHS), The University of Queensland, 20 Cornwall Street, WoolloongabbaAUS-4102, Queensland, Australia; ⊥ School of Mathematical Sciences, University of Adelaide, Ingkarni Wardli Building, North Terrace Campus, SA-5005Adelaide, Australia

## Abstract

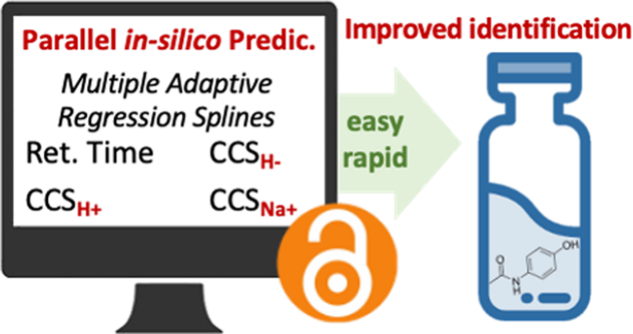

Ultra-high performance liquid chromatography coupled to ion mobility separation and high-resolution mass spectrometry instruments have proven very valuable for screening of emerging contaminants in the aquatic environment. However, when applying suspect or nontarget approaches (*i.e.*, when no reference standards are available), there is no information on retention time (RT) and collision cross-section (CCS) values to facilitate identification. In silico prediction tools of RT and CCS can therefore be of great utility to decrease the number of candidates to investigate. In this work, Multiple Adaptive Regression Splines (MARS) were evaluated for the prediction of both RT and CCS. MARS prediction models were developed and validated using a database of 477 protonated molecules, 169 deprotonated molecules, and 249 sodium adducts. Multivariate and univariate models were evaluated showing a better fit for univariate models to the experimental data. The RT model (*R*^2^ = 0.855) showed a deviation between predicted and experimental data of ±2.32 min (95% confidence intervals). The deviation observed for CCS data of protonated molecules using the CCS_H_ model (*R*^2^ = 0.966) was ±4.05% with 95% confidence intervals. The CCS_H_ model was also tested for the prediction of deprotonated molecules, resulting in deviations below ±5.86% for the 95% of the cases. Finally, a third model was developed for sodium adducts (CCS_Na_, *R*^2^ = 0.954) with deviation below ±5.25% for 95% of the cases. The developed models have been incorporated in an open-access and user-friendly online platform which represents a great advantage for third-party research laboratories for predicting both RT and CCS data.

## 1 Introduction

In the last decade, considerable effort has been devoted to enhance the performance of high-resolution mass spectrometry (HRMS) suspect screening (SS) and nontarget screening (NTS) strategies.^1−3^ The instrumental improvements of HRMS instruments have required the development of more sophisticated algorithms to be able to handle the large amount of data generated.^3,4^ Therefore, the development of open-access scripts for data processing and in silico prediction tools represents a step-forward into the applicability of SS and NTS in wide-scope campaigns by facilitating the identification process.^5−7^ Furthermore, the establishment of community-adopted levels of confidence for the identification of compounds using chromatography coupled to HRMS has been of paramount importance for the comparison of data across studies.^8^

Recently, ion mobility separation (IMS) coupled to HRMS instruments (IMS-HRMS) has proven promising for SS and NTS strategies.^9^ It permits, in theory, to resolve co-eluting compounds with the same nominal or exact mass that could not be previously separated with solely the chromatographic method, such as isobaric or isomeric compounds.^9−11^ Moreover, it allows the removal of mass spectrometric peaks that do not correspond to the feature of interest, which is particularly beneficial in data independent acquisition (DIA) experiments.^9,10,12^ As a consequence, there is a reduction in the necessity of data-dependent analysis because the full-spectrum HRMS acquisition can be filtered on both RT and ion mobility data.^12,13^

Collision cross section (CCS) values, derived from drift time (DT) measured by IMS, are known to be system- and matrix-independent and, therefore, experimental CCS data can be included in home-made or online databases with an expected deviation below 2% for most cases.^9,14,15^ However, this is not the case for absolute chromatographic retention times (RT) which cannot easily be compared between instrumental configurations even when RT indexing approaches are applied.^16−18^ Thus, reference standards are practically required for building a home-made database. However, SS and NTS strategies for the identification of emerging contaminants are commonly applied prior to the acquisition of the corresponding reference standards ^1,3^ and, therefore, lacking any information on experimental RT and CCS. In this sense, in silico prediction tools of either chromatographic retention data or ion mobility data are of great utility to decrease the number of candidates to investigate and, therefore, increase the chance of correct identification of features.^6^

Several studies have predicted RT,^6,19−28^ CCS values,^7,29−36^ or both.^13^ Predictors of RT have been developed mainly to model RT data in reverse-phase liquid chromatography (RPLC) and hydrophilic interaction liquid chromatography (HILIC) with prediction accuracy between approximately ±1 to ±3 min (up to 22% of the chromatographic gradient length). However, there is no clear agreement in the literature on how to express the prediction accuracy of the models or which should be the most appropriate statistical descriptor representing the prediction power of the system developed.^6^ Although CCS could be theoretically modeled from the three-dimensional and chemical structure using supercomputing systems,^34,37−39^ data-driven predictive models have also been developed showing predictive accuracies in the range of 3–6% for Traveling Wave Ion Mobility instruments (TWIMS)^29,31,32,35^ and Drift Tube Ion Mobility instruments (DTIMS).^30,31,33^ Similar prediction accuracy was obtained by Mollerup et al. in their study for the simultaneous prediction of RT and CCS.^13^ However, these data-driven models were fed with data generated using different instruments depending on the output parameter. For the RT prediction, they used data gathered from an ultra-high performance liquid chromatography (UHPLC)-HRMS instrument, while for CCS prediction, they modeled CCS data generated with a UHPLC-IMS-HRMS instrument. Because RT variations could probably be observed across instruments, the utility of predicted RT in the identification of UHPLC-IMS-HRMS features is limited.

In general, the reported models were based on univariate or multivariate regressions,^24,35^ artificial neural networks (ANNs),^13,22,25,29,31^ quantitative structure-retention relationships (QSRR),^6,21,40^ supported vector regression (SVR),^30,33,36^ or statistical analysis.^32,35^ Although Multivariate Adaptive Regression Splines (MARS) have been previously explored for RT prediction, there has been no prior exploration of the simultaneous prediction of RT and CCS.^41,42^ MARS is a multivariate nonparametric regression procedure that was first proposed by Friedman.^43^ One of the biggest advantages of MARS compared to the “black box” methods of ANNs is that they yield a straightforward model with simple quadratic relationship and, therefore, they are easy to interpret, with the interactions between variables clearly indicated.^41^ Additionally, the developed MARS models for predicting analytically relevant parameters requires limited informatics resources and knowledge of prediction software tools and can consequently easily be performed. In this sense, MARS has previously been applied in the chemical sciences for quantitative structure-retention relationships.^41,44^ However, the application of MARS for the combined prediction of chromatographic and ion mobility data of emerging contaminants has not previously been evaluated and reported in the literature.

In this work, a prediction model for both RT and CCS has been developed using MARS for the identification of candidates in SS and NTS strategies using UHPLC-IMS-HRMS. To facilitate other laboratories implementing this predictive tool in their workflows, a free online-available application has been released. This is, to best of the authors knowledge, the first application of MARS for the prediction of RT and CCS data. Additionally, it is the first parallel RT and CCS predictive model for the same instrument facilitating the identification process of emerging contaminants in SS and NTS strategies.

## 2 Materials and Methods

### 2.1 Chemicals and Materials

A set of 556 reference standards encompassing illicit drugs, hormones, mycotoxins, new psychoactive substances, pesticides, and pharmaceuticals was injected for the development of a CCS and RT library.^9^Table S1 of the Supporting Information shows the complete set of compounds used in the study with their SMILES (simplified molecular-input line-entry system) representation and measured RT and CCS data. This database is also available on the *Zenodo* online repository.^45^ Within this data set, 477 protonated adducts ([M + H]^+^), 169 deprotonated adducts ([M – H]^−^), and 249 sodium adducts ([M + Na]^+^) were used for the development and validation of the CCS predictive models.

### 2.2 Instrumentation

Retention time and CCS data were obtained with a Waters Acquity I-Class UPLC system (Waters, Milford, MA, USA) coupled to a VION IMS-QTOF mass spectrometer (Waters, Milford, MA, USA), using an electrospray ionization (ESI) interface operating in positive and negative ionization mode and following the method presented in Celma et al.^9^

The chromatographic column used was a CORTECS C18 2.1 × 100 mm, 2.7 μm fused core column (Waters) at a flow rate of 300 μL min^–1^. Gradient elution was performed using H_2_O (A) and MeOH (B) as mobile phases, both with 0.01% formic acid. The percentage of B was initially set to 10%, and it was immediately linearly increased to 90% over 14 min, followed by a 2 min isocratic period, and then returned to initial conditions (at 16.1 min) with a 2 min equilibration of the column. The total run time was 18 min. The injection volume was 5 μL.

A capillary voltage of 0.8 kV and cone voltage of 40 V were used. The desolvation temperature was set to 550 °C, and the source temperature to 120 °C. Nitrogen was used as drying and nebulizing gas. The cone gas flow was 250 L h^–1^ and desolvation gas flow of 1000 L h^–1^. The column temperature was set to 40 °C and the sample temperature to 10 °C. MS data were acquired using the VION in HDMSe mode, over the range *m/z* 50–1000, with N_2_ as the drift gas, an IMS wave velocity of 250 m s^–1^, and wave height ramp of 20–50 V. Leucine enkephalin (*m/z* 556.27658 and *m/ z* 554.26202) was used for mass correction in positive and negative ionization modes, respectively. Two independent scans with different collision energies were acquired during the run: a collision energy of 6 eV for low energy (LE) and a ramp of 28–56 eV for high energy (HE). A scan time of 0.3 s was set in both LE and HE functions. Nitrogen (≥99.999%) was used as collision-induced dissociation (CID) gas. All data were examined using an in-house built accurate mass screening workflow within the UNIFI software (version 1.9.4) from Waters Corporation.

### 2.3 Retention Time and Collision Cross-Section Modeling

#### 2.3.1 Molecular Descriptors

A total of 1666 molecular descriptors were downloaded from Dragon v5.4 integrated within OChem website (Online Chemical Database with modeling environment, www.ochem.eu).^46^ The complete set of descriptors for the molecules used in the study is available in Table S1.

#### 2.3.2 Prediction Model

MARS analysis was applied to predict both RT and CCS for protonated adducts ([M + H]^+^) in a single multivariate model. Additionally, univariate models for individual RT and CCS for protonated adducts ([M + H]^+^) (CCS_H_) and sodium adducts ([M + Na]^+^) (CCS_Na_) were also performed. Because of the expected low correlation between RT and CCS (*r = 0.354*), a multivariate model was not considered essential. As a further justification for this decision, the cross-validated *R*^2^ values for the multivariate model were 0.798 for RT and 0.964 for CCS_H_. This suggests instability on the data that is varying the accuracy of the model fits (particularly for RT). Therefore, the development of a multivariate MARS model able to predict simultaneously RT and CCS simultaneously was discarded.

MARS was able to select the most suitable molecular descriptors for each model (Table 1), and predictive interval bands were constructed for the univariate cases assuming a linear model variance structure. To meet this assumption, the square root of RT was modeled. The selection of molecular descriptors was automatically performed by the MARS algorithm during the model development, so no chemical bias from the analysts would influence the method.

**Table 1:** Descriptors Needed for Each of the Univariate MARS Models for RT and CCS_H_ and CCS_Na_^a^^,^^b^

molecular descriptors		
RT	CCS_H_	CCS_Na_
ALOGP	AMR	AMR
ALOGPS_LogP	L1m	Har2
BEHm4	LPRS	MAXDN
GATS1m	MDDD	Mor17m
Mor16m	nRCHO	nR09
N-068	PCR	piID
nDB	Whetp	QXXv
nRNHR		QZZm
O-059		RDF065v
STN		ZM1v

a Note that there are no similarities between the three univariate models.

b *ALOGP*: Ghose-Crippen octanol–water partition coefficient (logP) (calculation based on Viswanadhan et al.;^49^*ALOGPS_LogP*: Ghose-Crippen octanol–water partition coefficient (logP) (calculation based on Tetko and Tanchuk;^50^*AMR*: Ghose-Crippen molar refractivity; *BEHm4*:highest eigenvalue n. 4 of Burden matrix/weighted by atomic masses; *GATS1m*: Geary autocorrelation – lag 1/weighted by atomic masses; *Har2*: square reciprocal distance sum index; *L1m*: 1st component size directional WHIM index/weighted by atomic masses; *LPRS*: log of product of row sums; *MAXDN*: maximal electrotopological negative variation; *MDDD*: mean distance degree deviation; *Mor16m*: 3D-MoRSE – signal 16/weighted by atomic masses; *Mor17m*: 3D-MoRSE – signal 17/weighted by atomic masses; *N-068*: Al3-N atom-centered fragment; *nDB*: number of double bonds; *nR09*: number of 9-membered rings; *nRCHO*: number of (aliphatic) aldehydes; *nRNHR*: number of secondary (aliphatic) amines; *O-059*: Al-O-Al atom-centered fragment; *PCR*: ratio of multiple path count over path count; *piID*: conventional bond-order ID number; *QXXv*: Qxx COMMA2 value/weighted by atomic van der Waals volumes; *QZZm*: Qzz COMMA2 value/weighted by atomic masses; *RDF065v*: radial distribution function – 6.5/weighted by atomic van der Waals volumes; *STN*: spanning tree number (log); *Whetp*: Wiener-type index from polarizability weighted distance matrix; *ZM1v*: first Zagreb index by valence vertex degrees.^51^

The CCS_H_ prediction model was also explored for the prediction of CCS for deprotonated adducts ([M – H]^−^) and sodium adducts ([M + Na]^+^). CCS_H_ accurately modeled [M – H]^−^ data but could not predict data at acceptable levels of accuracy for [M + Na]^+^. Therefore, an exclusive univariate model was considered for the prediction of CCS data for sodium adducts (CCS_Na_).

All analyses were complete using *R*,^47^ and MARS analysis was completed using the earth package with the variance structure defined using the linear model (lm) option.^48^

## 3 Results and Discussion

### 3.1 Development and Validation of Prediction Models

#### 3.1.1 Individual RT and CCS Model Development

There is no assumption of an underlying variance structure with the multivariate MARS analysis, and there was no facility to define one within the earth package at the time of implementation. However, for the univariate analyses, a linear model variance structure was defined. This meant the standard deviation was estimated as a function of the predicted response and, hence, allowed for the construction of prediction intervals.

It is essential to use prediction intervals, rather than confidence intervals, in cases where the goal is to predict future values. A prediction interval is wider than a confidence interval and, at the 95% level, will provide bounds within which 95% of predicted values should fall.

All analyses considered the whole set of 1666 molecular descriptors as possible inputs to be used in the models. The assumptions of normality, linearity, and homoscedasticity were assessed for the univariate models which held those assumptions. The univariate MARS fit to RT violated the assumptions of linearity and homoscedasticity, so a square root transform was applied. This then reasonably met assumptions.

In summary, three different univariate models were developed for the prediction of RT (eq 1), CCS data for (de)protonated molecules (CCS_H_) (eq 2), and CCS data for sodium adducts (CCS_Na_) (eq 3). As an example and to assist with interpretation, in eq 1, the term 0.099·max(0,(nDB-3)) is equal to 0 for nDB ≤ 3 and equal to 0.099·(nDB-3) for nDB > 3.

RT model (eq 1):
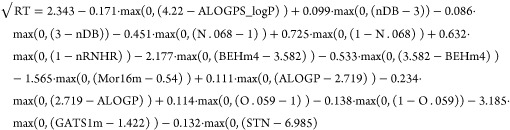
1

CCS_H_ model (eq 2):
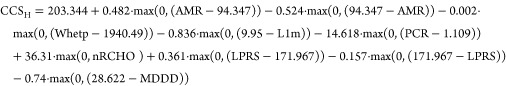
2

CCS_Na_ model (eq 3):
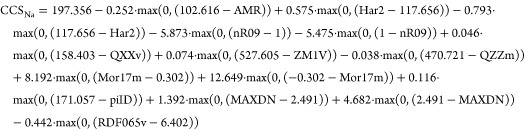
3

The univariate models obtained a cross-validated *R*^2^ = 0.855 for the RT model, *R*^2^ = 0.966 for the CCS_H_ model, and *R*^2^ = 0.954 for the CCS_Na_ model. Table 1 reveals that the univariate models (RT and CCS_H_) do not share a single descriptor, lending weight toward the argument that univariate models provide better fits to the data than the previously explored multivariate model.

#### 3.1.2 Cross-Validation of the RT, CCS_H_, and CCS_Na_ Models

MARS models were fitted using a threefold cross-validation with 30 iterations. This procedure splits the data into three sections, fits the model to two of those sections (*training data*), and then tests the accuracy of the resulting model on the final section (*test data*). This procedure is then repeated 30 times, each time randomly dividing the data into three sections. The measure of accuracy used to assess goodness of fit is the cross-validated *R*^2^, which looks at the average *R*^2^ value obtained across all 30 iterations when the model was fit to the test data. This value is usually lower than the *R*^2^ for the best model fit but dramatic changes suggest volatility in the data or overfitting in the modeling procedure.

**Figure 1:**
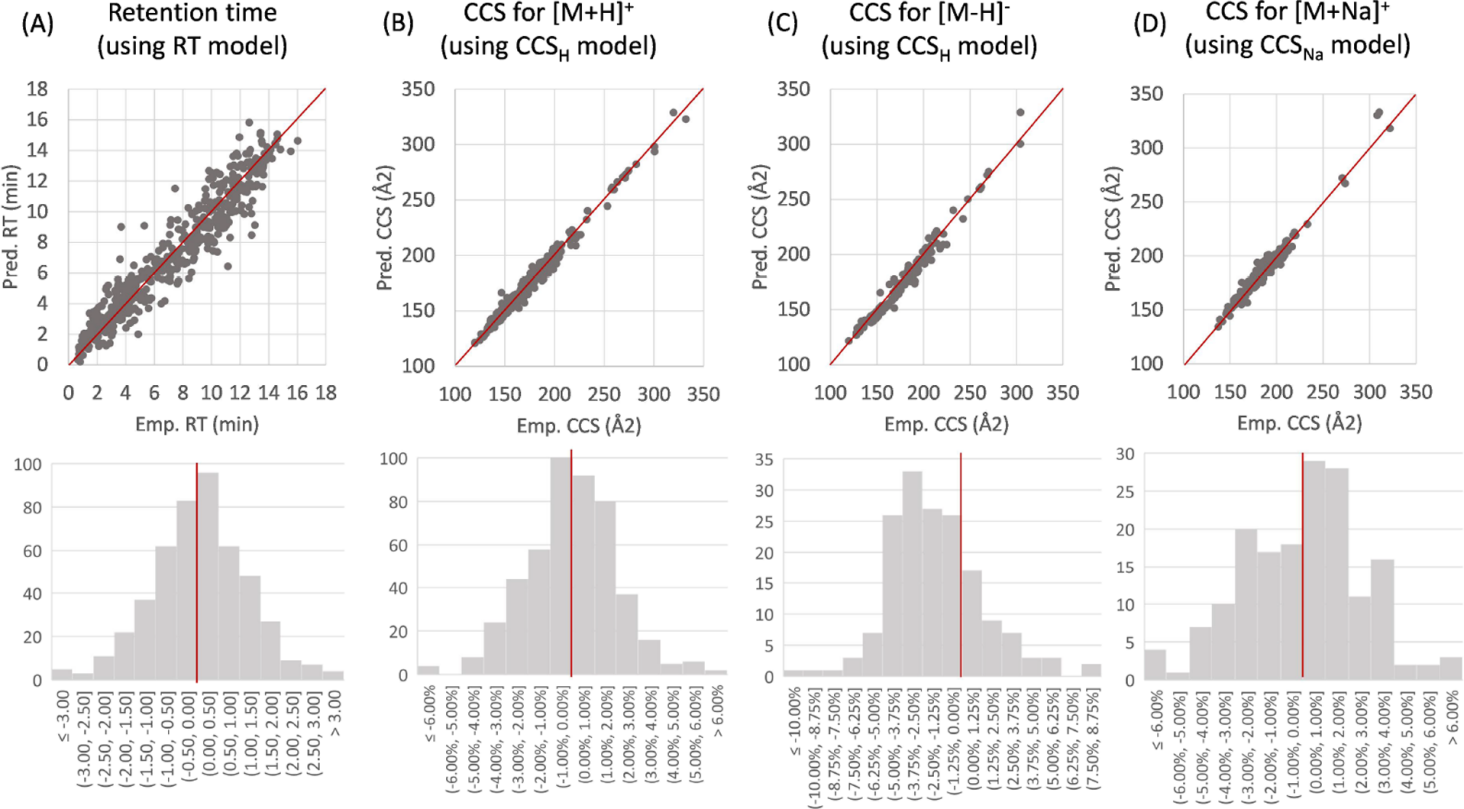
*Top:* Comparison of experimental and predicted RT data using the RT model (A), CCS for protonated molecules using CCS_H_ model (B), CCS for deprotonated molecules using the CCS_H_ model (C), and CCS for sodium adducts using the CCS_Na_ model (D). (Red line indicates region where Experimental CCS = Predicted CCS) *Bottom:* Histogram distribution of deviations between experimental and predicted data for RT data using the RT model (A), CCS for protonated molecules using the CCS_H_ model (B), CCS for deprotonated molecules using the CCS_H_ model (C), and CCS for sodium adducts using the CCS_Na_ model (D). (Red vertical lines indicate 0% deviation).

**Figure 2:**
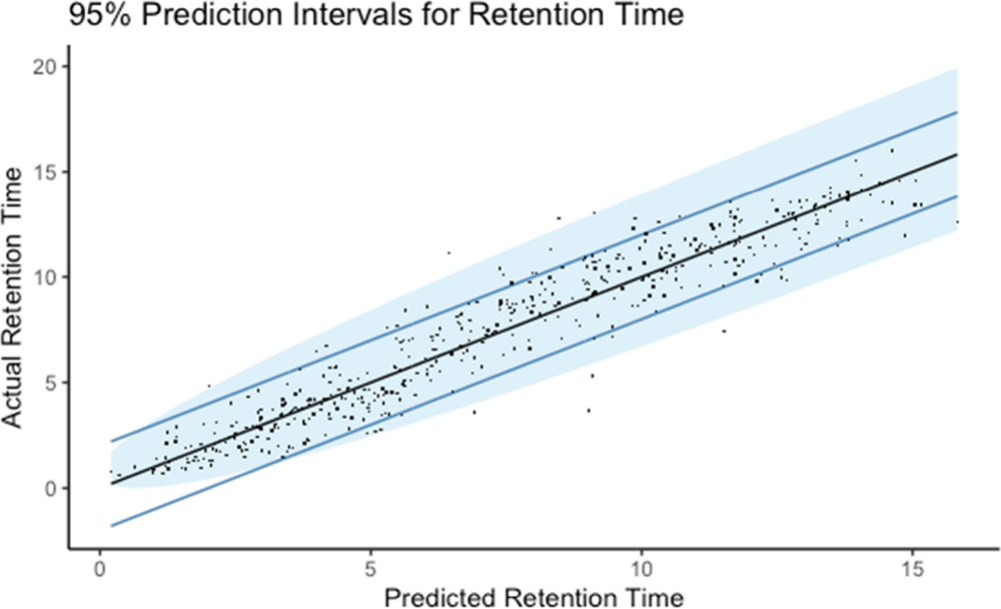
95% prediction intervals (blue area) for the univariate MARS analysis on the square root of RT. Blue lines are placed at the predicted values ±2 min. Approximately, only 8% of observed retention times were more than 2 min away from their predicted value.

In order to perform an additional model evaluation and to obtain an overview of the model performance, RT and CCS data were predicted for the molecules used for model development, but flagged as “unknown” substances. By comparing predicted and experimental RT data (Figure 1A, top), it was observed that the average deviation obtained using the RT model (eq 1) was ±0.72 min, as shown in Table 2. However, 95% of the predictions fell within ±2.32 min. Additionally, it could also be observed that deviations in predicted data distributed normally around 0% deviation (marked as a red line in Figure 1A, bottom) The prediction accuracy obtained is an improvement for the 95% intervals in previously developed models (±4.0 min (22%) using linear correlation *logKow* predictor,^24^ ±2.80 min (15%) using ANNs^25^ over a total chromatographic run of 18 min) and in line with the model developed by means of ANN by Mollerup et al. (over ±2 min (13%) deviation in a total run of 15 min).^13^ The developed model herein presented also improves the prediction accuracy compared to Barron and McEneff where they obtained an average deviation of ±1.02 min^22^ (3–13% for the gradient length ranging 8–35 min). As another way of presenting prediction accuracy, Figure 2 plots the predicted vs experimental data with the 95% prediction intervals (blue area) for the univariate MARS analysis of the . Approximately, only 8% of predicted RT were more than 2 min away from experimental ones. In this figure, we can also observe the 95% interval boundaries of the predicted values. This should be estimated depending on the RT because the prediction intervals are not constant across the whole chromatogram.

Prediction accuracy for CCS data was also studied. The deviation observed for CCS data of [M + H]^+^ using CCS_H_ model averaged ±1.23%, being ±4.05% within 95% of the cases (Table 2). Figure 1B, bottom shows that deviations randomly distributed around 0% (marked as a red line) value without biasing predicted data. When compared with previous models, the CCS_H_ model outstands the performance of developed ANNs prediction tools for CCS data of protonated molecules, which showed an accuracy of ±5–6% for 95% of the cases^13,29^ or roughly ±2.5% deviation for 50% of the cases.^31^ This vast improvement in the accuracy could be explained by the larger database used for the model development as well as the better fitting of experimental data with MARS than ANNs. In addition, the present method also improves other machine learning models, such as CCSbase, which yield an accuracy slightly over ±5% deviation (95% confidence interval).^32^ The recently developed model AllCCS used more than 5000 experimental CCS values to train a support vector regression-based prediction model, which resulted in an accuracy of ±4% for 84% of the cases.^36^ The obtained accuracy is in line with that obtained in the present study, although the CCS_H_ model is slightly more accurate for predictions because 95% of the cases have a deviation of ±4.05%.

**Figure 3:**
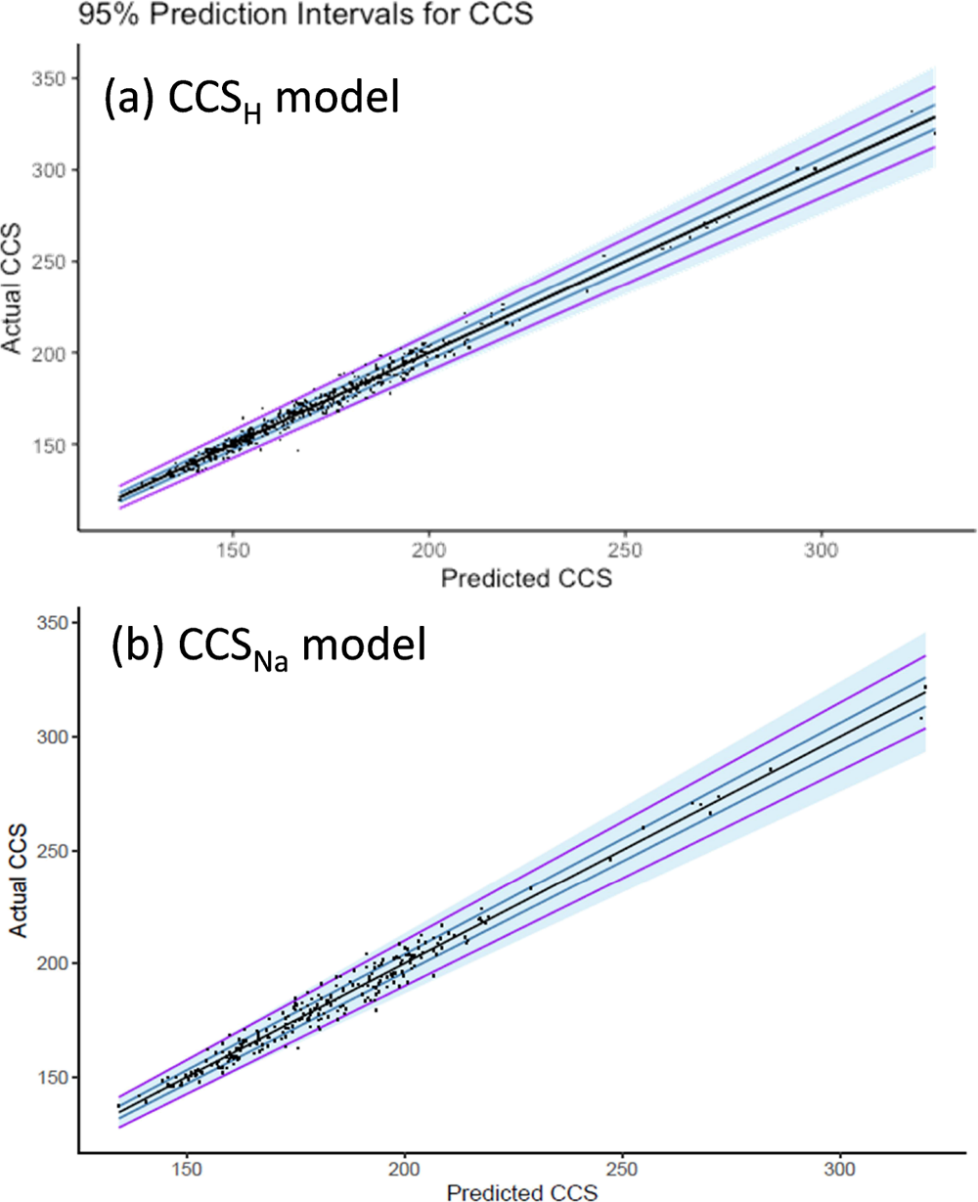
95% prediction intervals (blue area) for the univariate MARS analysis on (a) CCS_H_ and (b) CCS_Na_ models. blue lines are placed at 2% error bands and the purple at 5%. It is clear that the model still predicts well at higher values where there are less data but the prediction intervals are much larger to accommodate the uncertainty due to lack of data.

Figure 3A shows the 95% prediction intervals (blue area) for the univariate MARS analysis on the CCS_H_ model. The blue lines are placed at predicted values ±2 Å^2^ and the purple are ±5 Å^2^. It is clear that the model still predicts well at higher values because all data points are below the purple lines. However, because there are less data at higher CCS values, the prediction intervals are much larger to accommodate the uncertainty than they are in the low CCS values range where there are more data, resulting in a better fit.

Additionally, the application of the CCS_H_ model for the prediction of CCS values for deprotonated molecules was tested, yielding highly accurate predictions (Figure 1C, top). By predicting mobility data for a set of 169 molecules ionized in negative mode, it was observed that the differences between the observed and predicted CCS for the [M – H]^−^ fell, 95% of the time, within −13.4 and 9.3 Å^2^, with a slight tendency to under-predict CCS values (Figure 1C, bottom). In relative terms, average deviation for [M – H]^−^ data was ±2.79% (±5.86% for the 95% of the cases, Table 2). Although these deviations seem larger than the ones observed for [M + H]^+^ data, this increase in the deviations observed for [M – H]^−^ was expected because the model was developed with [M + H]^+^ data. However, it was assumed that the predictions of the CCS_H_ model developed with [M + H]^+^ data could also be extrapolated to the prediction of CCS data for [M – H]^−^, as no remarkable improvement was expected if a model was exclusively developed for deprotonated molecules.

Ideally, a unique model for the prediction of CCS for (de)protonated molecules and sodium adducts was intended. Therefore, the CCS_H_ model was also tested against [M + Na]^+^ data. However, high deviations were observed (±4.77% average, ±10.86% for the 95% of the cases, Table 2), which could be expected because of the likely higher impact of the volume of the sodium atom in the overall CCS of the molecule. In light of these data, [M + Na]^+^ data required a separate model for CCS prediction that was different to the one initially developed. The procedure for the CCS_Na_ model development was equivalent to the process described above (section 2.3) but using as input a data set of 249 CCS values for [M + Na]^+^ ions. The accuracy of the model was evaluated by also comparing predicted and experimental data (Table 2). Prediction deviations were ±2.08% on average (±5.25% for the 95% of the cases), showing a great improvement compared with predicted data using the CCS_H_ model. Figure 3B depicts the predicted vs experimental CCS values comparing the 95% prediction intervals (blue colored area) for the univariate MARS analysis on the CCS_Na_ model. The fact that different predicted values can be obtained for both protonated molecules and sodium adducts is of great help for experimental observations of both species for a suspect substance. Hence, increased confidence on the tentative identification can be garnered by matching both of the CCS values observed with the predicted data.

**Table 2:** Deviations at Percentiles 50 (Average), 95, and 99 for the Predicted RT and CCS Data during Model Validation

model		average deviation	deviation at 95%	deviation at 99%
RT		±0.72 min	±2.32 min	±3.82 min
CCS_H_	[M + H]^+^	±1.23%	±4.05%	±6.33%
	[M – H]^−^	±2.79%	±5.86%	±8.39%
	[M + Na]^+^	±4.77%	±10.86%	±12.80%
CCS_Na_	[M + Na]^+^	±2.08%	±5.25%	±6.86%

The CCS_Na_ model herein presented also improves the prediction accuracy of the previously developed model by the authors.^29^ In that work, we evaluated the performance of the ANN predictive model for sodium adducts, finding that deviations between predicted and experimental data were below 8.7% for 95% of the cases. However, the development of an exclusive model for the sodium adducts by MARS improves the prediction accuracy.

#### 3.1.3 Blind Testing of the Models

Several reference standards were purchased from different research projects during the development of the predictors based on MARS. However, these newly available compounds were not included in the training and validation data sets used but were used to verify the utility of our prediction models for chemicals not previously considered in the training steps. Thus, model applicability can be extrapolated for upcoming RT and CCS predictions of real suspect compounds. Therefore, we calculated deviations between predicted and experimental data for this data set and compared the observed deviations with previously calculated accuracies at different percentiles (shown in Table 2). Table 3 depicts the experimental and predicted values of RT and CCS for the different adducts observed for the additional set of 25 reference standards. Moreover, the deviation between experimental and predicted is shown, and as it can be observed, the RT predictions are generally in agreement with the experimental data with the 95th percentile of the observed deviations (±4.15 min) being in the same range than that observed during validation. However, *diphenyl hydrogen phosphate* showed a high deviation between experimental and predicted values. This can be potentially explained because of the lack of sufficient compounds featuring a P atom in their chemical structures in the initial training database. Hence, it is not surprising that for these molecular skeletons, the RT prediction does not fit precisely with the experimental data.

**Table 3:** Experimental and Predicted Values of RT and CCS for Additional Compounds Not Initially Included in Data Sets: Investigation of the Deviation of Predicted Values

compound	retention time (min)			CCS_H_ for [M + H]^+^			CCS_Na_ for [M + Na]^+^			CCS_H_ for [M – H]^−^		
	exp.	pred.	dev (min)	exp.	pred.	dev (%)	exp.	pred.	dev (%)	exp.	pred.	dev (%)
(−)-cotinine	0.87	3.05	2.18	141.48	136.12	–3.79%						
3,4-dichloroaniline	7.92	6.21	–1.71	137.10	125.87	–8.19%						
3-hydroxyphenyl diphenyl phosphate	11.09	10.36	–0.73	174.31	178.94	2.66%	184.84	189.67	2.61%	180.89	178.94	–1.07%
5,6-dimethylbenzotriazole	6.74	4.38	–2.36	129.73	127.21	–1.94%				129.38	127.21	–1.68%
8-hydroxyquinoline	1.51	4.57	3.06	125.01	123.54	–1.18%						
amisulpride	2.46	1.99	–0.47	193.15	189.60	–1.84%						
antiblaze V6	10.84	14.49	3.65	208.45	207.32	–0.54%	208.45	212.12	1.76%			
benzotriazole	3.50	2.75	–0.75	121.49	117.94	–2.92%						
BClPHP phosphate^a^	8.07	7.69	–0.38	159.22	157.05	–1.36%	165.14	166.24	0.67%			
caffeine	3.08	1.94	–1.14	136.62	136.37	–0.18%						
chlorotoluron	2.54	6.79	4.25	146.29	146.00	–0.20%	155.35	157.96	1.68%			
citalopram	6.49	5.21	–1.28	179.10	184.48	3.01%						
Di(2-ethylhexyl) terephthalate	16.86	15.02	–1.84				216.36	197.07.23	–8.61%			
diphenyl hydrogen phosphate	12.46	5.06	–7.41	152.45	151.61	–0.55%	161.58	162.65	0.66%	152.18	151.61	–0.38%
diphenylcresyl phosphate	7.36	11.07	3.72	175.28	178.08	1.60%						
metolachlor ESA^b^	7.89	4.99	–2.90	168.38	171.29	1.73%	175.57	179.13	2.03%	174.30	171.29	–1.73%
metoxuron	5.98	7.04	1.06	149.83	150.62	0.53%	158.51	161.17	1.68%			
mono(2-ethylhexyl) phthalate	12.73	11.75	–0.98							170.91	167.767215	–1.84%
monuron	6.68	5.67	–1.01	140.59	142.94	1.67%						
nicotine	0.69	1.11	0.42	138.34	134.77	–2.58%						
niflumic acid	11.51	10.86	–0.65	157.46	157.79	0.21%				156.92	157.79	0.55%
pirbuterol	1.30	1.28	–0.02	153.78	156.91	2.04%	160.02	165.52	3.44%			
prometon	6.74	7.50	0.76	156.67	155.56	–0.71%						
trietazine	10.81	8.91	–1.90	150.63	151.12	0.33%						
vildagliptin	1.38	1.67	0.29	176.98	174.62	–1.33%	172.29	187.35	8.74%			


a Bis(1-chloro-2-propyl) 1-hydroxy-2-propyl phosphate.

b Metolachlor ethane sulfonic acid.

Furthermore, the vast majority of CCS values for [M + H]^+^ are in agreement with the values calculated using the CCS_H_ model. For these compounds, 95% of the cases showed deviations below ±3.71%, yielding even better results than the initial database during model validation. Only *3,4-dichloroaniline* shows a deviation greater than 4%, which could be explained by the small CCS value calculated. When evaluating CCS_Na_, higher deviations are observed concretely for the case of *di(2-ethylhexyl) terephthalate* and *vildagliptin* (−8.61 and 8.74%, respectively). These deviations could be explained because of the particular chemical structures of the molecule such as the presence of an adamantyl group in *vildagliptin*, which has a large and rigid structure, or the high rotatability of alkyl chains in the *di(2-ethylhexyl) terephthalate*. However, if these adducts would be treated as outliers, 95% of the CCS_Na_ values show deviations of ±3.15%, which is in accordance with the data obtained during method validation. Finally, for [M – H]^−^, a small set of molecules was gathered, and all of them fit well within the ±5.8% deviation.

### 3.2 Open-Access Prediction Platform

**Figure 4:**
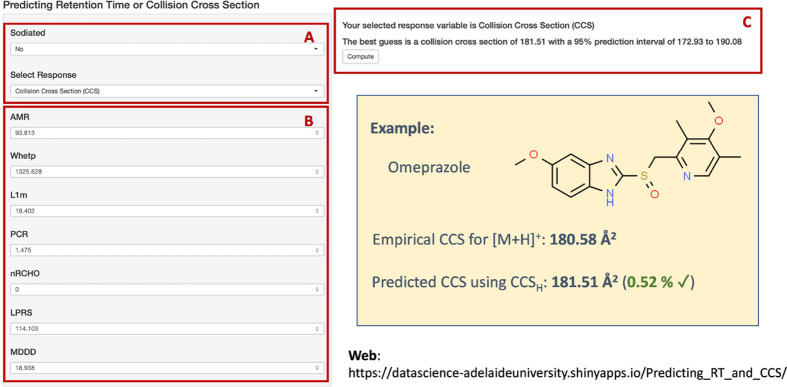
Online platform for the prediction of RT and CCS data using univariate models. (A) Selection of response to predict, that is, RT, CCS for (de)protonated molecules or CCS for sodium adducts; (B) introduction of molecular descriptors for the molecule of interest; (C) output of the predictor model together with the prediction intervals. Example illustrated by omeprazole.

To aid future researchers working with UHPLC-IMS-HRMS, a free online webpage incorporating these models has been released. The models are available for the scientific community through https://datascience-adelaideuniversity.shinyapps.io/Predicting_RT_and_CCS/. Figure 4 illustrates the layout of the online platform for the prediction of RT and CCS for both (de)protonated molecules or sodium adducts.

The operational of the platform is user-friendly and easy-to-follow. As an example, the step-by-step method to obtain prediction for omeprazole is shown. First, selection of which parameter is going to be predicted need to be done (Figure 4A). In this case, CCS for protonated molecules is selected by indicating “Select Response: Collision Cross Section” and “Sodiated: No”. After downloading the appropriate descriptors for the molecule of interest using Dragon v5.4 integrated within OChem (www.ochem.eu),^46^ those can be added in the corresponding editable fields (Figure 4B). The CCS value can, then, be predicted, and the output is shown together with their corresponding prediction intervals (Figure 4C). In this case, the CCS predicted value for the protonated molecule of omeprazole is 181.51 Å^2^ with a prediction interval of 171.93–190.08 Å^2^. The experimental value for [M + H]^+^ for omeprazole is 180.58 Å^2^, denoting that the prediction only deviated by 0.52% from the experimental value.

The ease of prediction as well as the open access for this online platform is of great help for those researchers working on UHPLC-IMS-HRMS instruments who do not have an in-house-developed prediction model.

## 4 Conclusions

Three different prediction models using MARS have been developed for the prediction of RT, CCS for (de)protonated molecules, and CCS for sodium adducts. This is the first application of MARS for the prediction of RT and CCS data. In addition, the reported models are the first parallel prediction of RT and CCS data for the same instrument, facilitating the identification process of chemicals of emerging concern in SS and NTS strategies. The developed predictive models make use of a set of 26 molecular descriptors to predict RT and/or CCS values. The prediction accuracy achieved with these models bettered previously reported models in the literature by reducing the deviation between predicted and experimental to ±2.32 min for RT, ±4.05% for CCS of protonated molecules, ±5.86% for CCS of deprotonated molecules, and ±5.25% for CCS of sodium adducts (95% confidence intervals). Additionally, a free access online platform has been released to enable the application of these models to third-party laboratories interested in predicting RT and CCS data.

## Data and Software Availability

Data used for model development and validation are available in Table S1 of the Supporting Information as well as on the open-access online repository Zenodo (https://zenodo.org/record/3966751#.Ymf5f9rP1aQ, DOI: 10.5281/zenodo.3966751). Additionally, molecular descriptors are also shown in Table S1. Mathematical equations resulting from MARS model development are available for their implementation throughout the manuscript (eqs 1–3).

## Supporting Information Available

Whole database of substances used for model development and validation including RT, CCS values, as well as the set of 1666 molecular descriptors (XLSX)
